# Correlation between asthma control and quality of life in patients with moderate and severe asthma

**DOI:** 10.1186/1939-4551-8-S1-A139

**Published:** 2015-04-08

**Authors:** Mary Jane Lisboa Valory, Norma Rubini, Albertina Varandas Capelo, Eliane Miranda Da Silva, Fernando Samuel Sion, Carlos Alberto Morais De Sa

**Affiliations:** 1 Federal University of the State of Rio De Janeiro, Brazil

## Background

The evaluation of the degree of disease control and quality of life of patients with asthma is a challenge for general practitioners and medical specialists. The aim of this study was to assess the degree of disease control and quality of life in patients with moderate and severe asthma in clinical follow-up of Allergy and Immunology clinic as well as comparatively evaluate two instruments for assessing asthma control, versus quality of life evaluation.

## Methods

We conducted a prospective, cross-sectional study from March 2011 to July 2013, including patients with moderate or severe persistent asthma in regular clinical monitoring. To assess the degree of asthma control we used the Asthma Control Test (ACT) and the criteria established by the Global Initiative for Asthma (GINA). In evaluating quality of life (QoL) we employed a specific questionnaire (Juniper et al, 1992). Descriptive and comparative statistical analysis was performed and p> 0.05 considered significant.

## Results

We evaluated 64 patients with moderate or severe persistent asthma, with ages between 18 and 82 years (mean ± SD = 53.42 ± 42.13), with 94% female, 64% classified as moderate persistent asthma and 36% as severe asthma and atopy present a frequency of 92%. According to the ACT, 58% of the patients had controlled asthma and 42% uncontrolled asthma, while in the GINA criteria, 53% of patients were classified as having controlled asthma, 16% partially controlled asthma and 31% uncontrolled asthma. In the comparative analysis between the assessment by the ACT and GINA there was no statistically significant difference (p> 0.05). In analyzing quality of life, 45% of the patients rated their quality of life as satisfactory. The present study showed a good agreement between the evaluations of the ACT and GINA, when compared to the quality of life questionnaire, with a concordance, respectively 84% and 85% (p> 0.05).

## Conclusions

We concluded that about 50% of the patients had partially controlled or uncontrolled asthma, with significant impact on quality of life. We observed a high concordance in the assessment of asthma control through ACT and GINA criteria, as well as a good agreement between the assessment of asthma control and quality of life. The results of this study reinforce the importance of routine use of assessment instruments for asthma control and quality of life in clinical practice.

